# Methodological Considerations about the Use of Bimodal Oddball P300 in Psychiatry: Topography and Reference Effect

**DOI:** 10.3389/fpsyg.2016.01387

**Published:** 2016-09-21

**Authors:** Elisa Schröder, Hendrik Kajosch, Paul Verbanck, Charles Kornreich, Salvatore Campanella

**Affiliations:** Laboratory of Psychological Medicine and Addiction, ULB Neuroscience Institute, CHU Brugmann – Université Libre de BruxellesBrussels, Belgium

**Keywords:** event-related potentials, bimodal P300, P3a, P3b, topography, reference

## Abstract

Event-related potentials (ERPs) bimodal oddball task has disclosed increased sensitivity to show P300 modulations to subclinical symptoms. Even if the utility of such a procedure has still to be confirmed at a clinical level, gathering normative values of this new oddball variant may be of the greatest interest. We specifically addressed the challenge of defining the best location for the recording of P3a and P3b components and selecting the best reference to use by investigating the effect of an oﬄine re-reference procedure on recorded bimodal P3a and P3b. Forty young and healthy subjects were submitted to a bimodal (synchronized and always congruent visual and auditory stimuli) three-stimulus oddball task in which 140 frequent bimodal stimuli, 30 deviant “target” stimuli and 30 distractors were presented. Task consisted in clicking as soon as possible on the targets, and not paying attention to frequent stimuli and distractors. This procedure allowed us to record, for each individual, the P3a component, referring to the novelty process related to distractors processing, and the P3b component, linked to the processing of the target stimuli. Results showed that both P3a and P3b showed maximal amplitude in Pz. However, P3a displayed a more central distribution. Nose reference was also shown to give maximal amplitudes compared with average and linked mastoids references. These data were discussed in light of the necessity to develop multi-site recording guidelines to furnish sets of ERPs data comparable across laboratories.

## Introduction

Event-related potentials (ERPs) are of strong interest in psychiatry. They consist of a change in the electroencephalogram (EEG) indexing the neural processing of a stimulus. Thanks to their high temporal resolution, they can give a wide outlook at information processing in normal and/or pathological subjects (Hansenne, 2006). Indeed, analyses of different ERPs may allow the assessment of many stages of cognitive treatment, thanks to earlier components [such as sensory gating P50 or mismatch negativity (MMN)] but also to later components (P300 or N400; Rugg and Coles, 1995). Therefore, it is possible through ERPs to study cognitive functions and processes of psychopathological conditions by comparing clinical populations to matched healthy controls (Polich and Herbst, 2000). However, up to this day, only some ERPs of interest in psychiatry benefit from detailed guidelines regarding the recommended recording and analysis parameters (Duncan et al., 2009). Therefore, there is still a crucial need for the development of multi-sites guidelines as the *a priori* choice of parameters such as the reference or the electrodes of interest defined by topography may be a source of bias introduced by the experimenter (Murray et al., 2008). Those choices can thus impact the statistical outcomes and the interpretation of the data. With this in mind, this paper addresses a first overview of some recording and analysis parameters for the bimodal P3a and P3b on healthy participants in a task implementable in psychiatry.

Since their discovery (Walter et al., 1964; Desmedt et al., 1965; Sutton et al., 1965), ERPs raised a hope to contribute to the elaboration of differential diagnosis for mental diseases (Boutros et al., 2011) as they were considered as potentials biological markers of psychopathology. Among others, one component was mainly expected to play the role of psychophysiological marker of psychiatric disorders: the P300 (Hansenne, 2006). The P300 is a parieto-central positive wave occurring roughly 300 ms after the presentation of a stimulus. It appears when a subject consciously detects an informative task-relevant stimulus (Huang et al., 2015). More specifically, the P300 appears to be the neural reflection of a revision process of mental representations in working memory, induced by the apparition of a stimulus: when a stimulus appears, it is compared to representations available in working memory. If a change is detected, the representations in working memory are updated thanks to attentional processes which are concomitant to the P300 (Polich, 2007). The P300 is usually recorded during an oddball paradigm in which two types of stimuli are presented: one frequent occurring around 80% of trials and one deviant, occurring around 20% of trials. Subjects have to detect the deviant target stimuli, typically by pressing a button or by mental counting.

Many studies were interested in P300 as potential state, trait and vulnerability markers in disorders such as schizophrenia, depression, and alcoholism. Schizophrenia is, by far, the most studied pathology in regards with ERP analysis. The P300 has been shown to be impaired in schizophrenia as schizophrenic patients display a less ample P300 than controls. The difference seems to persist whether the patient is in an acute phase or in remission, which suggests the P300 is a trait marker of schizophrenia (see Mathalon et al., 2000 for review). Regarding depression, results are more equivocal, some studies on depressive patients in acute phase suggest a reduced P300 amplitude is associated with a longer latency while other studies failed to replicate those results (Hansenne, 2006). Heterogeneity of the findings is probably due to the heterogeneity of the psychiatric population itself, since different subgroups of depressive patients display different kind of P300 alterations (the P300 seems to be influenced by suicidal risk, anxiety or the presence of psychotic symptoms; Hansenne, 2006). Despite those variations, the reduction of P300 amplitude is considered as a state marker of depression as its amplitude tends to increase during the treatment (e.g., Anderer et al., 2002). On the other hand, alcoholic patients exhibit a reduced P300 amplitude (e.g., Maurage et al., 2007) but this finding seems to be also true for children of alcoholic parents (e.g., Polich et al., 1994), which suggests this decrease in amplitude might play a role of vulnerability factor in alcoholism.

The P300 was first regarded as a potentially useful tool in diagnosis but happened to be mostly useful as an index of cognitive performances, as it provides physiological measures associated with attentional engagement and memory operations in task (Polich, 2004, 2007). Because the latency of an ERP component is thought to reflect the speed of processing and the amplitude is viewed as the amount of resources allocated to the task (Hansenne, 2006), the analysis of the P300 might be of great help in order to assess cognitive abilities of a patient. However, the clinical use of the P300 is very low, mostly due to its tremendous variability and to its lack of sensitivity (Hansenne, 2006; Mathalon et al., 2010). With this consideration, attempts were made to strengthen the oddball paradigm in order to generate a more sensitive P300. Campanella et al. (2010) suggested oddball paradigm might be lacking of sensitivity since it is administrated in a single sensory modality (visual or auditory). Indeed, in everyday life, sensory events are multimodal and integrated into a unitary perception thanks to higher level integrative processes. Hence, they created a bimodal oddball paradigm in which stimuli were presented simultaneously in an auditory and visual modality. This “bimodal” task proved to be more sensitive to subclinical groups, as subjects with anxiety and depressive tendencies exhibited lower P300 amplitudes compared to controls in the bimodal oddball task only. Those findings have been confirmed in emotional paradigms (Campanella et al., 2010) and neutral conditions (Campanella et al., 2012; Delle-Vigne et al., 2014). Moreover, the discriminative power of the P300 has also been observed on other (sub)clinical populations, as in subjects with alexithymia (Delle-Vigne et al., 2014). This suggests that P300 might still be an indicator of a difference in the neural process of subjects presenting subclinical tendencies but only when confronted with an adapted, more sensitive and more ecological task.

Overall, even if these data still have to be extended to clinical populations, the bimodal P300 oddball design disclosed up to now preliminary encouraging results, pointing to an increased sensitivity of the P3b to subclinical differences as compared to unimodal classical conditions. Therefore, two additional points seem to be important to be faced with: (i) the promotion of multi-site guidelines to record electrophysiological measures that may be compared and used across studies is primordial, as this could help to avoid functional misinterpretations of the data as well as to prevent the emergence of controversial results from different laboratories (Campanella and Colin, 2014); and (2) the P3a or novelty P3 is of high interest in psychiatry, as it has been shown to be more sensitive than the classical P3b to depression (e.g., Bruder et al., 2009), alcoholism (e.g., Hada et al., 2000) and psychosis (e.g., Atkinson et al., 2012). The best way to measure the P3a is with a three-stimulus type oddball task including frequent stimuli, deviant targets, and distractors (Polich and Criado, 2006). Distractors are referred to novel and unexpected stimuli that the subjects have to ignore, occurring at the same frequency as deviant target stimulus. Those novel stimuli trigger a positive brain potential maximal at fronto-central areas occurring between 250 and 550 ms, the P3a, which is thought to be the marker of orientation of attention (Knight, 1996). In this way, including the P3a in the evaluation of psychiatric populations through the P300 appears as a priority in ERPs research in psychiatry. The main aim of the present paper is then triple: first, we tested the implementation of a third type of stimulus (distractors) in a very simple bimodal task usable in psychiatry in order to measure the P3a in addition of the P3b classically referred as P300; second, we investigated which electrodes appear to be the most well-suited to register the bimodal P3a and P3b components the most accurately; and finally, we also investigated which reference electrode appears as the most adapted to the recording of a bimodal P3a and P3b.

Indeed, to our knowledge, no paradigm ever attempted to measure a bimodal P3a, whose sensitivity could also benefit from a bimodal procedure in the image of bimodal P3b. Therefore, we created a visual × auditory bimodal oddball task with three types of stimulus: frequent, deviant target, and distractors. Young and healthy subjects underwent the procedure, allowing us to explore the implementation of the P3a in a bimodal paradigm. In the same way, addressing the challenge of selecting the best reference for the recording of the bimodal P3a and P3b is essential, as the activity on reference sites affects measurements at all active electrode sites (Yao et al., 2007). To the best of our knowledge, this question has never been addressed before in a bimodal task as no recording recommendations exist for this very new paradigm. In this view, we investigated the reference effect through an oﬄine re-reference procedure. Amplitudes and latencies of the P3a and P3b were measured and compared through average, nose, and linked mastoids references.

## Materials and Methods

### Participants

Forty young and healthy participants were recruited through a dedicated platform on a social network for this study. Most of them were students at our university. Subjects had to be 20–30 years old. Exclusion criteria, assessed during a short anamnesis, were as follow: previous or current neurological problem such as head trauma and epilepsy; previous or current psychiatric disorder including suspicion of an addictive disorder other than smoking; current medical treatment that could affect cognitive performances; and uncorrected visual or auditory deficiency. Group characteristics are reported in **Table 1.**

**Table 1:** Demographic and clinical characteristics.

	Mean	Standard deviation
Age (years)	24.29	3.03
Education (years)	15.71	2.36
Alcohol consumption (drinks per week)	6.94	6.23
Tobacco consumption (cigarettes per day)	3.47	6.93
Depression (Beck score)	8.03	6.82
Anxiety (State) (STAI-A score)	45.29	8.30
Anxiety (Trait) (STAI-B score)	48.38	8.83
Impulsivity (UPPS score)	93.65	19.96

### Oddball Task

The task consisted of a bimodal (visual and auditory) oddball paradigm. Participants were confronted with three types of stimuli: frequent standard stimulus, rare target (deviant stimulus), and rare non-target stimulus (distractor). Frequent stimulus consisted of a woman face combined with a woman voice pronouncing the French word “papier,” deviant target consisted of a man face along with a man voice pronouncing the French word “papier” and non-target distractors consisted of a picture of an animal along with his call (six different animals) for one half of the sample. For the other half, frequent stimuli consisted of the man face and deviant target stimuli of the women face. Subjects were asked to click as quickly as possible on a button with their right hand for each deviant target stimulus and to ignore any other stimuli. They were not informed that distractors would be presented.

**FIGURE 1:**
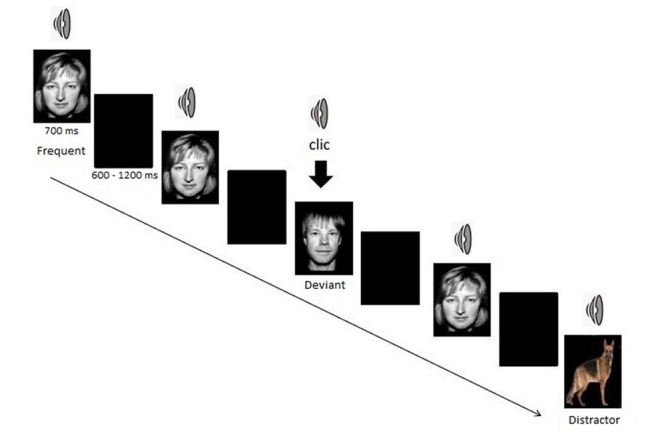
**The bimodal oddball task**.

The task was presented in one block of approximately 10 min, consisting of 140 frequent stimuli (70%), 30 deviant target stimuli (15%), and 30 distractors (15%). Each picture was presented for 700 ms. A black screen was displayed between pictures for a random duration of 600–1200 ms (**Figure 1**).

Response times and percentages of correct answers were recorded.

### Procedure

The local ethics committee at the Brugmann Hospital (“Comité d’Éthique Hospitalier OM 026”) approved our study. Informed written consent was first obtained from each participant. They received an explanation of the nature and duration of the study and were informed of what was expected from them. They were told that they were free to participate or not, as well as to leave the study at any time without having to justify their decision.

The experiment started with the oddball task: participants sat in a darkened room on a chair approximately 1 m from a computer screen. Following the task, participants were asked to complete the following questionnaires: the State-Trait Anxiety Inventory (STAI-A and STAI-B; Spielberger and Gorsuch, 1983); the Beck Depression Inventory (BDI-II; Beck et al., 1987); and the Urgency Premeditation Perseverance and Sensation seeking impulsive behavior scale (UPPS; Whiteside and Lynam, 2003), as anxiety, depression and impulsivity are personality factors known to induce P300 modulations, even at a subclinical level (e.g., Rossignol et al., 2005, 2008; Fritzsche et al., 2011). Finally, a short anamnesis assessing alcohol and drugs consumption, neurological and psychiatric history, and education was administrated. They were paid 25 Euros for their full participation.

### EEG Recording and Analysis

During the testing phase, EEG activity was recorded with 33 electrodes mounted on a Quik-Cap and placed in standard (based on the 10–20 system) and intermediate positions. Recordings were made with a common average, re-references were computed oﬄine (nose and linked mastoids). The EEG was amplified with battery-operated ANT^®^ amplifiers with a gain of 30,000 and a bandpass of 0.01–100 Hz. The impedance of all electrodes was maintained below 10 kΩ. EEG was recorded continuously at a sampling rate of 1024 Hz with ANT Eeprobe software. 99.9% of the participants’ responses were correct (i.e., a finger tap given for deviant target stimuli). Only correct answers were considered for analysis of reaction times and EEG activity. The trials contaminated by eye movements or muscular artifacts were manually eliminated oﬄine and discarded from further analyses. The number of included trials in subsequent statistical ERP analyses was of 24.32 (/30) ± 4.67 for the novelty P3 and of 25.85 (/30) ± 3.55 for the P3b. Epochs starting 200 ms before the onset of the stimulus and lasting for 800 ms were created (Bruder et al., 2009; Duncan et al., 2009). The data were filtered with a 30 Hz low-pass filter. To compute averages of P3a/P3b to distractors/target stimuli for each subject, two parameters were coded for each stimulus: (i) the type of stimulus (frequent; deviant target; distractor); and (ii) the type of response [correct responses: keypress for deviant target stimuli and no keypress for frequent stimuli and distractors; errors: no keypress for deviant targets (omissions) or keypress for frequent stimuli and distractors (commissions)]. A general time window was first determined globally for the identification of the components of interest (P3a and P3b) based on the literature. The measurement window was then tailored for each participant: for each subject, both P3a and P3b were investigated by gathering individual values of maximum peak amplitudes and peak latencies for each stimulus type in a 300–600 ms time range (Comerchero and Polich, 1998, 1999; Duncan et al., 2009). These data were obtained on the following electrodes: Fz, F3, F4, FC1, FC2, Cz, Pz, P3, P4, and Oz. Measurements were gathered for P3a and P3b with three references: common average (A), nose (Nz), and linked mastoids (Lke).

### ERP Statistical Analysis

We analyzed ERP and behavioral data using analysis of covariance (ANCOVA) and analysis of variance (ANOVA). Our aims were to investigate the topography of P3a and P3b on a bimodal oddball task as well as the effect of the reference used on those two ERPs. Four levels (Fz, Cz, Pz, and Oz) within subjects ANCOVAs were first performed on amplitudes of P3a and P3b separately for each reference to evaluate the midline topography of those components with personality variables (STAI-A, STAI-B, UPPS, and BDI-II) as covariables. As those variables had no effect on the results (all *p* > 0.05), we removed them from the analysis and conducted our analysis as ANOVAs. Secondly, three levels (A, Nz, Lke) within subjects ANOVAs were computed for P3a and P3b to evaluate which reference displays the best results on peak amplitudes. Finally, 2 × 4 within subjects ANOVAs were performed in order to compare the topography of the P3a vs P3b on each reference. Latencies of P3a and P3b at the site of interest were compared in each reference condition. Greenhouse–Geisser correction was applied to all ANOVAs when necessary. Bonferroni *post hoc t*-tests were used to explore interactions effects. All analyses were conducted with SPSS 20.00, with the level of significance at 0.05.

## Results

Among the 40 participants, six had to be excluded of the analysis: two presented clinical signs of a psychiatric disorder (assessed during the short anamnesis) and four displayed a bad signal-to-noise ratio (three had a number of averaged trials inferior to 10 for one component at least and one subject presented a signal contaminated by alpha waves). At the individual level, novelty P3 and P3b were visible for each remaining participant.

### Behavioral Results

The average performance on target detection was of 29.97 (/30) ± 0.17 with an average reaction time of 450 ± 45 ms. Subjects made on average 0.029 ± 0.17 omissions and 0.118 ± 0.33 commissions errors (pressing on a stimulus other than deviant target).

### Topography of P3a and P3b

**FIGURE 2:**
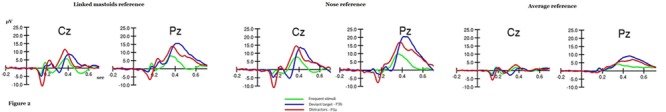
**Original P3a, P3b, and frequent stimuli waves at Cz and Pz sites, for linked mastoids, nose, and average references**.

Four levels within subjects ANOVAs (Fz, Cz, Pz, and Oz) on amplitude showed a significant electrode effect for P3a and P3b with the average, nose, and linked mastoid references (all *p* < 0.001). Bonferroni *post hoc t*-tests specifically showed that P3b had its maximal amplitude at Pz for all three references used. Similarly, P3a also displayed its maximal amplitude at Pz for all three references. We compared the topography of P3a and P3b with a 2 × 4 levels within subjects ANOVA (P3a and P3b × Fz, Cz, Pz, and Oz). Results showed a significant principal effect of electrodes for nose and linked mastoids references (*p* < 0.001 for Nz and Lke references, *p* = 0.077 for A reference) and a significant interaction effect (*p* < 0.001). Bonferroni *post hoc* showed that P3a had a more parieto-central distribution while P3b had a more strict parietal distribution (see **Figure 2**). Results are displayed in **Table 2.**

**Table 2:** Average amplitude (in microvolts) on Fz, Cz, Pz, and Oz electrodes for P3a and P3b, regarding nose (Nz), linked mastoids (Lke), and average references.

		Fz		Cz		Pz		Oz	
		Mean	Standard deviation	Mean	Standard deviation	Mean	Standard deviation	Mean	Standard deviation
Nz	P3a	11.55	6.63	15.69^∗^	7.10	18.22^∗∗∗^	6.86	12.28	7.64
	P3b	9.83	6.12	16.52	8.22	23.10 ^∗∗∗^	8.39	15.99	7.59
Lke	P3a	8.32	7.66	12.48^∗∗^	7.25	14.96^∗∗∗^	6.05	8.63	5.49
	P3b	5.95	8.29	11.99	9.01	18.18^∗∗∗^	7.92	10.73	4.63
Average	P3a	0.36	4.02	3.86	3.45	5.87^∗∗^	3.02	2.15	5.64
	P3b	-1.91	4.78	3.02	4.64	8.83^∗∗∗^	3.80	4.33	5.11

*^∗∗∗^Different from all other electrodes at the level of *p* < 0.001. ^∗∗^Different from all other electrodes at the level of *p* < 0.01. ^∗^Different from all other electrodes at the level of *p* < 0.05.*

Regarding the latencies of P3a and P3b, we calculated an average score for latencies of P3a and P3b on parietal electrodes (P3, Pz, and P4). Paired Student’s *t*-tests were computed on those average scores, showing that latencies of P3a and P3b were significantly different for nose [*t*(33) = -4.953; *p* < 0.001] and linked mastoids [*t*(33) = -5.741; *p* < 0.001] but not for the common average reference [*t*(33) = -0.927; *p* = 0.361].

### Reference Effect

A three levels within subjects ANOVA (Lke, Nz, A) on the mean amplitude score calculated on parietal electrodes (P3, Pz, and P4) showed a significant reference effect both on P3a and P3b (all *p* < 0.001). More precisely, Bonferroni *post hoc t*-tests showed that, for P3a and P3b, the nose reference displayed the highest amplitude (all *p* ≤ 0.001), followed by the linked mastoids reference, itself displaying higher amplitudes than the average reference (all *p* < 0.001; see **Figure 2**).

## Discussion

The main goal of this study was triple: (i) to implement a third type of stimulus, the distractors, in the bimodal oddball task in order to generate the P3a component; (ii) to test which electrodes generate maximal P3a and P3b amplitudes; and (iii) to specifically test the effect of different references on bimodal P3a and P3b recording. This study has first enabled us to determine the topography of bimodal P3a and P3b. Results showed that both P3a and P3b are best measured at parietal sites. P3a and P3b displayed a different topography as P3b appeared as a parietal wave occurring around 400 ms while P3a was defined as a more precocious parieto-central wave, occurring around 370 ms. Regarding the reference effect, amplitudes were significantly higher with a nose reference than a linked mastoids or a common average reference. Moreover, the differential distribution of P3a vs P3b was better shown with nose and linked mastoids references.

Taken together, these data furnished important methodological considerations. First, we were able to create a rather short task (10 min) allowing us to easily measure in healthy subjects both bimodal P3a and P3b by adding distractors in a classical two-stimulus type oddball task. The parietal nature of the P3b component in this task is consistent with findings in unimodal paradigms, as P3b seems to occur as a consequence of attentional resource activations promoting memory operations in temporal–parietal areas (Huang et al., 2015). On the other hand, P3a is often described as a component with a more fronto-central topography than the parietal-maximum P3b (Polich, 2007) given that P3a is thought to be the reflection of frontal attention mechanisms (Bruder et al., 2009), as it appears to be related to neural changes in the anterior cingulate cortex (Huang et al., 2015). While our results showed a more central distribution compared with P3b, they still suggest a parietal-maximum topography for P3a as well. This finding raises different questions: is the recorded component really a P3a that has the particularity to have a more parietal topography in bimodal oddball paradigm or is the recorded brain activity for distractors more a reflection of context updating in working memory, and therefore should be considered as a P3b? First, we observed that the latencies of our P3a were significantly shorter than the latencies for P3b. Since the P3a is believed to precede P3b (Kayser and Tenke, 2006), this observation goes in the favor of a generated P3a. Second, fronto-central P3a is often recorded in unimodal paradigms for which it is observed that an auditory task generates higher amplitudes in Fz and Cz while a visual task generates higher amplitudes in Pz and Cz locations (Comerchero and Polich, 1998). In this way, we cannot exclude the idea that a P3a generated in a bimodal visual × auditory oddball task could induce higher amplitudes in parieto-central electrodes. Third, the topography observed in our results matches the one obtained by Polich and Comerchero (2003) in an “easy task.” The authors manipulated the degree of perceptual distinction between the deviant target and the frequent stimulus, creating an “easy condition” and a “hard condition” (with the target physically similar to the frequent stimulus). In regard with the P3a, in the easy condition, the distractors elicited a parietal maximal P300, with a shorter latency and smaller amplitude than the P3b generated by the deviant targets while the P3a generated in the hard condition was larger, more central, and had a shorter latency. This suggests that the processing of distractors is affected by the discrimination task difficulty. As our task was built to be of simple use with psychiatric patients, the discrimination difficulty might be too low for control subjects to generate a fronto-central maximal P3a.

Second, analyses of the reference effect on P3a/P3b amplitudes favor the use of a nose reference in bimodal oddball paradigm. However, the literature emphasizes on the use of an average reference in unimodal paradigm (Duncan et al., 2009). The use of the average reference is of great interest in the search of a “closer-to-neutral” reference. Indeed, given the very nature of EEG recording, based on a potential difference, this technique is in need of a point of reference whose activity will affect measurements at all active reference electrode sites (Yao et al., 2007, 2005). In this way, an ideal reference electrode should deliver an activity constant over time and independent of the activity recorded at active electrodes (Bertrand et al., 1985). The average reference has therefore been considered as a suitable candidate, based on theoretical arguments suggesting that the integral of the potential distribution over a sphere including current dipoles is null (Bertrand et al., 1985). However, the use of the average reference in our paradigm had the main effect to lower amplitudes and to lessen the topographic differences between P3a and P3b. As a broadly distributed ERP component, the P300 dominates the activity that is “subtracted out” by the referencing procedure. In this way, a common average could decrease its amplitude. Although the use of a nose reference seems less theoretically justified and more arbitrary, we should keep in mind that an inactive or silent recording site does not exist anywhere on the body and, therefore, any choice of reference is inevitably arbitrary (Kayser and Tenke, 2015a,b). With this consideration, we believe that the reference choice can be driven by the will to use a conventional standard which allows easy comparisons and emphasize some features of brain activity (Dien, 1998). This led, for instance, to the choice of a nose reference in the recording of the MMN, as it specifically allows the discrimination between the MMN and the N2b (Näätänen et al., 2007). Since the use of a common average reference seems to obscure some patterns of brain activity in the case of a visual × auditory cross-modal oddball paradigm, we believe the use of a nose reference would be the most appropriate.

Overall, main data of the present study are (1) P3a is maximally recorded at centro-parietal sites, with a latency around 375 ms, and a mean amplitude value of 17 μV with nose reference; while (2) P3b displays a maximal amplitude at parietal sites with a latency around 409 ms and a mean amplitude value of 22 μV with the nose reference. These methodological considerations are highly relevant to furnish a simple task that can be easily adapted in every labs to favor multi-site recordings (comparable data tagging exactly similar cognitive functions). Obviously, our study has also some limitations: we have a rather small number of participants, methodological considerations should therefore be very carefully taken on this sample as we were not able to control the influence of gender, age, education level and mostly personality parameters since our sample lacked of variability on those aspects (we tested only young and healthy subjects, most of them being students in our university). Our data are therefore preliminary and should be confirmed on bigger sample, controlling for the influence of the cited parameters. Another limitation is that we did not counterbalance the physical characteristics of our stimuli across all categories. Indeed, if male and female faces were counterbalanced between frequent stimuli and deviant targets, animal images and shouts were only used as distractors. This was done following previous research about the novelty P3 (Friedman et al., 2001; Polich and Comerchero, 2003; Bruder et al., 2009) in order to generate a novelty P3 of high amplitude with a very simple task, which can be critical when working with psychiatric patients. As the amplitude of the novelty P3 is directly correlated to the degree of novelty of a stimulus and to the physical characteristics of the stimuli (Gaeta et al., 2003; Polich and Comerchero, 2003), we used six different distractors stemming from environmental sounds to restrain the impact of repetition on the amplitude of the novelty P3 and to increase the probability of having a novelty P3 of high amplitude (Cycowicz and Friedman, 2004). However, future research regarding the general use of a bimodal three-stimulus oddball paradigm should control for the impact of the physical characteristics of the stimuli on the generated P3a and P3b. Finally, another limitation is that our subjects gave a motor response only for deviant target stimuli. While this is what is usually done in multiple studies with control subjects and psychiatric patients (Polich and Comerchero, 2003; Bruder et al., 2009; Campanella et al., 2010), this might be an issue as motor response has been shown to have an interference effect on recorded ERP parameters (Starr et al., 1997; Kotchoubey, 2014). A solution might be to ask to subjects to mentally count the targets instead of giving a motor response (Verleger, 1991). However, as the goal is for this task to be implementable in psychiatry, this solution could be too challenging for patients.

## Conclusion

Finding sensitive tools is one of the main current challenges in experimental psychopathology. In this way, advances were specifically made in the field of ERPs. For instance, the addition of the P3a to the classical P3b analysis in depression allowed a better discrimination between depressive and healthy controls (Bruder et al., 2009). Similarly, it was previously thought that early-phase psychotic patients did not display a constant diminution of MMN amplitude (Salisbury, 2012) but recent studies showed a higher sensitivity of the MMN when recorded during an adapted, more sensitive paradigm (Rudolph et al., 2015). Those studies all meet the same purpose of finding biological markers of psychiatric diseases as such markers might help to (1) index the recovery of a patient during the follow-up and (2) highlight cognitive dysfunctions in order to orient neurocognitive and/or neuromodulative remediations. This paper constitutes a first step toward the use of adapted paradigms in psychiatry, by offering a first glance of multi-site guidelines on bimodal P3a and P3b.

## Author Contributions

ES contributed to study design, data recording, analysis and interpretations, statistical analysis, and paper writing. HK contributed to the design and critically reviewed the paper. CK and PV critically reviewed the paper. SC was the principal designer of the study and contributed to its implementation, supervised data recording, interpretation, and paper writing.

## Conflict of Interest Statement

The authors declare that the research was conducted in the absence of any commercial or financial relationships that could be construed as a potential conflict of interest.
